# Eye Movements of French Dyslexic Adults While Reading Texts: Evidence of Word Length, Lexical Frequency, Consistency and Grammatical Category

**DOI:** 10.3390/brainsci15070693

**Published:** 2025-06-27

**Authors:** Aikaterini Premeti, Frédéric Isel, Maria Pia Bucci

**Affiliations:** 1 Modèles, Dynamiques, Corpus (MoDyCo), Centre National de la Recherche Scientifique (CNRS), Université Paris Nanterre, 92001 Nanterre, France; 2 Laboratoire de Phonétique et Phonologie, Institut de Linguistique et Phonétique Générales et Appliquées (ILPGA), Centre National de la Recherche Scientifique (CNRS), Université Sorbonne Nouvelle, 75012 Paris, France; fredericisel@gmail.com; 3 Interactions, Corpus, Apprentissages, Représentations (ICAR), Centre National de la Recherche Scientifique (CNRS), École Normale Supérieure de Lyon, Université Lumière Lyon 2, 69342 Lyon, France

**Keywords:** developmental dyslexia, reading aloud, eye-movements, phonological deficit, visuo-attentional deficit

## Abstract

Background/Objectives: Dyslexia, a learning disability affecting reading, has been extensively studied using eye movements. This study aimed to examine in the same design the effects of different psycholinguistic variables, i.e., grammatical category, lexical frequency, word length and orthographic consistency on eye movement patterns during reading in adults. Methods: We compared the eye movements of forty university students, twenty with and twenty without dyslexia while they read aloud a meaningful and a meaningless text in order to examine whether semantic context could enhance their reading strategy. Results: Dyslexic participants made more reading errors and had longer reading time particularly with the meaningless text, suggesting an increased reliance on the semantic context to enhance their reading strategy. They also made more progressive and regressive fixations while reading the two texts. Similar results were found when examining grammatical categories. These findings suggest a reduced visuo-attentional span and reliance on a serial decoding approach during reading, likely based on grapheme-to-phoneme conversion. Furthermore, in the whole text analysis, there was no difference in fixation duration between the groups. However, when examining word length, only the control group exhibited a distinction between longer and shorter words. No significant group differences emerged for word frequency. Importantly, multiple regression analyses revealed that orthographic consistency predicted fixation durations only in the control group, suggesting that dyslexic readers were less sensitive to phonological regularities—possibly due to underlying phonological deficits. Conclusions: These findings suggest the involvement of both phonological and visuo-attentional deficits in dyslexia. Combined remediation strategies may enhance dyslexic individuals’ performance in phonological and visuo-attentional tasks.

## 1. Introduction

Dyslexia is a neurodevelopmental disorder characterized by significant challenges in reading acquisition, despite adequate teaching and in the absence of any neurological or sensory deficits or below-average intelligence [1]. It affects approximately 5 to 10% of the population [2]. Individuals with dyslexia often experience difficulties in accurate and fluent reading, including substitutions and omissions, as well as challenges in word recognition, spelling, and decoding abilities.

The causes of dyslexia remain under debate. While the phonological deficit theory has traditionally been central—highlighting difficulties in grapheme-to-phoneme conversion and related issues with phonological awareness, memory, and word retrieval [3,4,5,6] other hypotheses have emerged (see the review of Stein [7]). These include deficits in auditory [8], and working memory [9], attention [10,11], magnocellular processing [12], and temporal oscillatory sampling [13]. The visuo-attentional deficit hypothesis also suggests a reduced ability to process several letters at once [10], which may underlie the atypical eye movements seen in dyslexia [14].

To complement these theoretical accounts, recent research has begun to examine how specific psycholinguistic variables affect reading behavior in dyslexia, particularly through real-time measures like eye tracking. Eye-tracking provides valuable, non-invasive insight into visual impairments in dyslexia by objectively capturing real-time reading behavior without relying on verbal responses or extra task demands [15,16]. Studies consistently show that individuals with dyslexia exhibit longer reading times, more frequent and unstable fixations, shorter forward saccades, and increased backward saccades in both children [17,18,19,20,21,22,23,24] and adults [25,26]. Additionally, research has found poor binocular coordination during and after saccades, as well as during prolonged fixations, in both reading and non-reading tasks [18,27,28,29,30,31]. For a review, see [32].

Specifically, studying eye movements is key to gaining insights that support early detection and effective rehabilitation for dyslexia-related reading difficulties. This approach also helps reveal how various psycholinguistic factors—such as word length, lexical frequency, consistency (i.e., the ambiguity in the correspondences between phonological and orthographic units of language [33]), grammatical category, and text complexity (at the level of meaning)—can significantly influence reading, especially in individuals with dyslexia.

Building on this, word length and frequency are well-established linguistic factors influencing reading and eye movements. Readers tend to fixate longer on long or infrequent words across languages (both in children [15] and in adults [34,35,36]) regardless of orthographic transparency—defined as the degree of consistency in mapping letter sequences to phonemes in both reading and spelling, classifying languages as transparent (shallow orthography) or non-transparent (deep orthography) [37]). In contrast, short [38,39] or highly frequent words [40,41] are often skipped. These effects are even more pronounced in individuals with dyslexia, particularly in children [19,22,42,43,44,45,46], but also in young adults [25,47].

Another important variable in word recognition is word consistency, which refers to the degree of correspondence between phonological and orthographic codes [33,48]. Consistency is highest (=1) when phoneme-grapheme mappings are unequivocal (e.g., table, chou in French), and decreases when multiple graphemes can represent a single phoneme (e.g., $/\tilde{\varepsilon }/$ →sain, pin, rein, un, in French) [49]. This parameter is crucial for word reading and is particularly relevant in dyslexia, where difficulties in grapheme-to-phoneme association are central. Research shows that visual word recognition is affected by inconsistency in both directions—spelling to phonology and phonology to spelling [50,51]. Such inconsistencies can hinder word recognition, fluency, and comprehension in individuals with dyslexia. Language differences also matter: English is inconsistent in both directions, while French is more consistent from orthography to phonology, but less so in reverse [51]. Studies using cross-linguistic eye-tracking paradigms—though still relatively scarce—consistently demonstrate that readers of more transparent orthographies (e.g., German) show stronger small-unit processing, whereas readers of deeper orthographies (e.g., English) rely more on large-unit processing. This expanded discussion highlights how language structure shapes eye-movement strategies and orthographic consistency effects [52]. For individuals with dyslexia, who already struggle with establishing consistent grapheme–phoneme associations, these cross-linguistic differences in orthographic depth may further amplify reading difficulties, making the influence of language structure particularly relevant when interpreting eye movement behavior.

In addition to consistency, processing word categories—particularly nouns and verbs—is a key aspect of language and reading, as they shed light on the cognitive underpinnings of grammatical distinctions [53]. Psycholinguistic research shows that nouns are generally processed faster than verbs across tasks such as lexical decisions [54,55,56,57,58], semantic categorization [58], and noun/verb classification [54,57] (for a review, see also [53]). This advantage likely stems from differences in syntactic, semantic, morphological, and formal properties between the categories.

Furthermore, semantic context in reading plays a vital role. A large body of research has demonstrated that meaningful context can strongly influence reading behavior, particularly through its effect on word predictability [59,60]. Eye-tracking studies have consistently shown that words that are semantically predictable from prior context are fixated for shorter durations, are fixated less frequently, and are more likely to be skipped altogether [61,62,63]. Understanding how semantic context guides eye movements is especially important in populations with reading difficulties, such as individuals with dyslexia, whose use of contextual cues may differ from that of typical readers.

Building on these findings, most prior research isolating psycholinguistic influences on eye movements—such as word length, frequency, category, and consistency—has focused exclusively on children or isolated word tasks. In contrast, our study integrates all these variables within a single, continuous text-reading paradigm. We further manipulate semantic context by having participants read two French texts: the meaningless L’Alouette [64] and the meaningful magazine excerpt Pollueur [65]. This design allows us to compare eye-movement patterns of dyslexic and control university students across both semantic conditions.

Our study investigates how four key psycholinguistic variables—word length, lexical frequency, grammatical category, and consistency of orthographic-to-phonological (and vice versa) mappings—affect eye movements during continuous text reading. Crucially, we compare dyslexic and control university students under two conditions: a meaningful and a meaningless text. This design allows us to see not only each variable’s individual impact but also how readers leverage—or fail to leverage—semantic context to anticipate upcoming words. By integrating all these factors into one paradigm, we aim to uncover the strategies dyslexic adults use and to contribute to the development of adaptive remediation strategies.

While much of the early research on dyslexia focused on children, a growing body of work in recent years has shifted attention to adults with developmental dyslexia. Studying adults provides valuable insights into the long-term trajectory of reading difficulties, revealing which impairments persist and which compensatory mechanisms may develop over time. This perspective is especially relevant when using eye-tracking methods, which have traditionally been applied more extensively to child populations. Examining eye movements in adult readers offers important information on how dyslexic individuals manage reading tasks after years of experience, and how their oculomotor behavior reflects both enduring difficulties and potential adaptations.

Our study aimed to examine this in a more ecologically valid reading situation—namely, natural reading contexts involving full texts. This design contrasts with much prior research that relied on isolated word or pseudoword presentations, or single-sentence reading, particularly in children. By embedding our psycholinguistic variables—word length, frequency, consistency, and grammatical category—within continuous texts, we can better understand how these factors interact during real reading. The two types of stimuli, a semantically meaningful text and a meaningless text, allow us to assess how context influences eye movement patterns in dyslexic adults, revealing whether they can benefit from top-down semantic cues in a manner similar to typically developing readers.

We hypothesize that, during continuous text reading, dyslexic participants will exhibit longer and more frequent fixations than controls, particularly on long or infrequent words, and that the effects of word consistency (orthographic to phonological and vice versa) will be attenuated in the dyslexic group. We further predict that semantic context will mitigate these differences: in the meaningful text, both groups should show reduced fixation durations and fewer regressions, whereas in the nonsensical text, dyslexic readers will fail to benefit from context, maintaining a pattern of increased fixations and regressions.

## 2. Materials and Methods

### 2.1. Participants

Twenty university students diagnosed with dyslexia (five men; mean age = 22 ± 3 years) matched in age with twenty control subjects (eight men; mean age = 22 ± 2.1 years) were included in the study. All participants were native French speakers with normal or corrected to normal vision (8/10 in each eye, according to Parinaud’s optometric scale [66]). Exclusion criteria included any known neurological disorders, comorbidities (such as ADHD or DCD), uncorrected visual impairments, or drug use. These were assessed through self-report via a screening questionnaire completed prior to participation. One initially recruited participant was excluded due to self-reported ADHD symptoms. The dyslexic participants were diagnosed by a speech therapist during childhood (mean age of diagnosis 7.3 ± 0.9 years) and have undergone several years of remediation (mean = 6.4 ± 3 years). All participants self-reported persistent reading and writing difficulties. Additionally, their current difficulties were evaluated using selected subtests from the ECLA 16+ battery to characterize the persistence of dyslexia-related impairments. All participants provided written informed consent prior to participating in the study, in accordance with the ethical standards of the relevant institutional research committee and the Declaration of Helsinki.

### 2.2. Screening Tests

Before the experiment, all participants underwent a battery of tests assessing their reading skills, phonological awareness, visuo-attentional skills, and non-verbal intelligence. Reading and reading-related skills (regular and irregular word reading and pseudoword reading, initial phoneme deletion, spoonerisms, non-word repetitions) were evaluated using the ECLA 16+ battery test [67]. Rapid automatized naming (RAN) was assessed with the ECLA 16+ subtest for letters, and adapted versions of the Phonological Assessment Battery [68] were used for figures and number naming. Visuo-attentional skills were evaluated using the a five-consonant global report task [10]. Nonverbal intelligence was assessed using the matrices and similarities subtests of the WAIS-IV [69]. Raw scores were used for reading, phonological, and RAN tasks. Standardized scores were used for the WAIS-IV subtests based on published norms. Independent-samples t-tests were applied to compare group performance across all screening measures, as summarized in Table 1. Several reading, phonological, and naming tasks demonstrated that the dyslexic group performed more than two standard deviations below the control group mean, including pseudoword reading (score and time), irregular word reading (time), regular word reading (time), initial phoneme deletion (score), spoonerisms (score and time), rapid automatized naming (RAN) for images, numbers, and letters (all time measures), and visuo-attentional span. This pattern is consistent with the expected diagnostic profile of dyslexia in adulthood and supports the persistence of reading and phonological difficulties beyond childhood.

### 2.3. Linguistic Material

The first text, L’Alouette [64], is a standardized reading test commonly used in France for the assessment of dyslexia. It consists of a grammatically correct but semantically meaningless text of 265 words. The second text, Pollueur, is a meaningful text taken from a magazine aimed at adolescents and young adults [65]. During the experiment, participants were asked to read aloud the first 16 lines of this text (231 words). In both texts, linguistic information concerning grammatical category, word frequency, word length (measured by number of letters) of each word was taken from the French database Lexique 3 [70]. Consistency refers to the degree of ambiguity between graphemes and phonemes and was treated as a continuous variable in this study, with values ranging from 0 (fully inconsistent) to 1 (fully consistent), based on statistical measures provided in the Lexique-Infra database [33].

In L’Alouette, the average word frequency was 1758.5 occurrences per million (SD = 5155.6), and the average word length was 5.1 letters (SD = 2.0). Grammatical category distribution was as follows: 33.5% nouns, 15.5% verbs, 9.6% adjectives, and 41.4% other categories (e.g., determiners, prepositions, and adverbs). In Pollueur, the average word frequency was 6164.0 (SD = 11047.7), with an average word length of 5.1 letters (SD = 2.8). The grammatical category distribution was 36.9% nouns, 18.4% verbs, 6.1% adjectives, and 38.5% other categories. The difference in word frequency is due to the nature of the Alouette text, which is composed of infrequent words in order to assess readers’ decoding skills independently of inference and anticipation strategies.

### 2.4. Eye Movement and Voice Recordings

All participants were tested individually in a soundproof room. They were seated 92 cm from a screen, with a chinrest and a forehead rest. Eye movements were recorded using the Eye-link 1000 eye tracker (Eyelink 1000 Desktop Mount, distributed by SR Research Ltd., Mississauga, ON, Canada). Before each session, nine-point gaze calibration was performed and repeated until the validation error was less than 1° on average and less than 1.5° at the worst point. Recording was conducted solely on the dominant eye of the participant, as there has been no observed relation between ocular dominance and reading skills [71,72].

Regarding text presentation, each full passage was displayed in its entirety on the screen throughout the reading task. Text was presented in black font on a white background to maximize contrast. The font type was Calibri, set at 18-point size. Lines were centered, with an average of 10 to 12 words per line and a line spacing of 1.5 times the font size to facilitate comfortable reading and natural eye movement. This layout aimed to resemble typical printed reading materials while ensuring visibility and minimizing visual strain. Ocular dominance was determined using the Miles test [73].

Fixations were assigned to specific words based on predefined interest areas (IAs) corresponding to each word’s spatial location on the screen. When fixations landed near word boundaries, a gaze-contingent mapping procedure was applied to refine fixation assignment and ensure accuracy. On average, 8.6% of trials were rejected for control participants and 10.0% for dyslexic participants due to quality issues such as calibration errors or blinks; this difference was not statistically significant (*p* = 0.139).

A Steinberg UR22 mkII microphone (Steinberg Media Technologies GmbH, Hamburg, Germany) was used for the voice registration. We have to note that voice recordings in the present study were only used for the measurement of reading time and of reading errors. Reading errors were identified based on the voice recordings. Errors included mispronunciations, omissions, and substitutions, and were only counted if the participant did not self-correct. Participants were instructed to read the text aloud as accurately as possible, avoiding errors, in a manner similar to how they would read in real-life situations. The instruction emphasized accuracy over speed, with the aim of promoting natural reading behavior and increasing the ecological validity of the task. The order of text presentation (Alouette and Pollueur) was counterbalanced across participants through random assignment. Half read the meaningless text first, the other half the meaningful text. No order effects were observed.

### 2.5. Data Analysis

The eye movement data were analyzed using Data Viewer software (version 4.2.1; SR Research Ltd., Kanata, ON, Canada). The number and duration of fixations were measured after both pro-saccades as well as retro-saccades. Additionally, saccade amplitude for both pro-saccades and retro-saccades was calculated. These eye movement parameters were also analyzed with respect to grammatical categories, specifically nouns, verbs, and adjectives and the lexical variables of the words (that is word frequency, number of letters, consistency of graphemes to phonemes, and consistency of phonemes to graphemes, both treated as continuous variables ranging from 0 to 1).

### 2.6. Statistical Analysis

Statistical analyses were conducted using JASP software (version 0.16.3.0, University of Amsterdam, Amsterdam, The Netherlands). The significance threshold was set at *p* < 0.05.

Independent-samples *t*-tests were used to compare participants with and without dyslexia on screening measures, including reading ability, reading-related skills such as phonological processing, visual-attentional span, and rapid automatized naming. Raw scores were used for these comparisons, except for IQ assessments, where standardized scores were analyzed.

To evaluate differences in reading behavior, we analyzed eight dependent variables: reading time (RT), number of errors, progressive fixation duration, number of progressive fixations, regressive fixation duration, number of regressive fixations and the amplitude of pro- and retro-saccades. Prior to analysis, Levene’s tests for equality of variances were conducted for all variables and revealed significant violations of homogeneity of variance in several cases. As a result, nonparametric tests were used throughout to ensure robust statistical inference. For each variable, we tested three effects: the main effect of Group (Control vs. Dyslexic), the main effect of Text (Alouette vs. Pollueur), and their interaction. The main effect of Group was assessed using independent-samples Mann–Whitney U tests on performance averaged across texts. The main effect of Text was assessed with Wilcoxon signed-rank tests comparing Alouette and Pollueur scores within participants. To assess Group × Text interactions, we computed difference scores (Δ = Alouette − Pollueur) for each participant and compared these across groups using Mann–Whitney U tests.

A second level of analysis using Repeated-measures ANOVAs was conducted separately for each dependent variable previously reported (reading time, reading errors, number and duration of fixations associated with a pro-saccade and a retro-saccade, and the amplitude of pro- and retro-saccades). The within-subject factor was Text (2 levels) and Grammatical Category (3 levels: noun, verb, adjective) was the within-subject factor alongside Text, with Group remaining the between-subject factor. When violations of sphericity were detected, the Greenhouse–Geisser correction was applied. Post hoc pairwise comparisons were performed using the Holm correction to control for multiple comparisons.

Moreover, Spearman’s rank-order correlation coefficients were computed by item (averaged across subjects) to investigate the relationships between the number and duration of fixations associated with both progressive and regressive saccades on each word, and four lexical variables: word frequency, number of letters, consistency of graphemes to phonemes, and consistency of phonemes to graphemes. To control for multiple comparisons, Bonferroni corrections were applied to adjust the significance threshold.

In addition to the correlations, we conducted multiple linear regressions to examine how word length, frequency, and consistency predicted fixation measures. Separate models were run for each group (dyslexic and control), and for progressive and regressive fixations. All predictors were centered. These analyses helped identify whether the lexical variables influenced eye movements differently in each group.

## 3. Results

### 3.1. Analyses on the Entire Text

Dyslexic participants had longer read times, made more errors, and showed increased numbers of fixations compared to controls, especially in the meaningless text (see also Table 2).

#### 3.1.1. Reading Time

**Figure 1:**
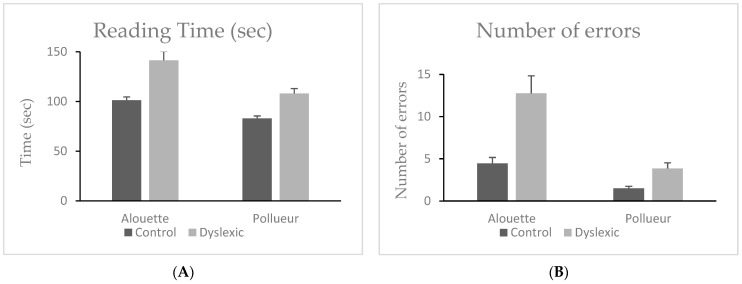
Mean reading time (**A**) and reading errors (**B**) during reading the “Alouette” (meaningless) and the “Pollueur” (meaningful) text in the group of control and dyslexic participants. Error bars represent standard errors.

A Wilcoxon signed-rank test showed that participants took significantly longer to read the Alouette text (M = 121.3 s, SD = 36.3) than the Pollueur text (M = 95.5 s, SD = 21.7), W = 820.00, z = 5.51, *p* < 0.001, ΔM = 25.8 s. A Mann–Whitney U test revealed that dyslexic readers (M = 124.7, SD = 30.9) had significantly longer reading times overall compared to control readers (M = 92.1, SD = 13.2), U = 53.50, *p* < 0.001, ΔM = 32.6 s. The group × text interaction was also significant, with the dyslexic group showing a larger increase in reading time between Alouette and Pollueur (M = 33.3, SD = 20.8) than the control group (M = 18.4, SD = 6.4), U = 89.00, *p* = 0.003, ΔM = 14.9 s (see Figure 1A). This result indicates that the effect of text difficulty—reflected by the longer reading times for the meaningless Alouette text—is more pronounced in dyslexic participants, underscoring the greater cognitive cost they experience due to reduced semantic predictability.

#### 3.1.2. Reading Errors

Participants made significantly more errors on Alouette (M = 8.6, SD = 8.1) than on Pollueur (M = 2.7, SD = 2.5), *W* = 732.00, *z* = 5.24, *p* < 0.001, ΔM = 5.9. Dyslexic participants made more errors overall (M = 8.3, SD = 5.9) than controls (M = 3.0, SD = 2.0), *U* = 41.00, *p* < 0.001, ΔM = 5.3. The interaction was also significant, with the dyslexic group showing a larger difference in errors between texts (M = 8.9, SD = 7.4) than the control group (M = 3.0, SD = 2.6), *U* = 75.00, *p* < 0.001, ΔM = 5.9. See also Figure 1B.

#### 3.1.3. Fixation Duration (Fixations Associated with a Pro-Saccade)

There was no significant difference in progressive fixation duration between texts (Alouette: M = 273.1 ms, SD = 45.2; Pollueur: M = 271.8 ms, SD = 40.6), *W* = 534.00, *z* = 1.67, *p* = 0.097. No group difference was found between dyslexic (M = 271.1, SD = 43.4) and control participants (M = 273.9, SD = 34.3), *U* = 219.00, *p* = 0.620. The group × text interaction was also not significant, *U* = 184.00, *p* = 0.678.

#### 3.1.4. Number of Fixations (Fixations Associated with a Pro-Saccade)

**Figure 2:**
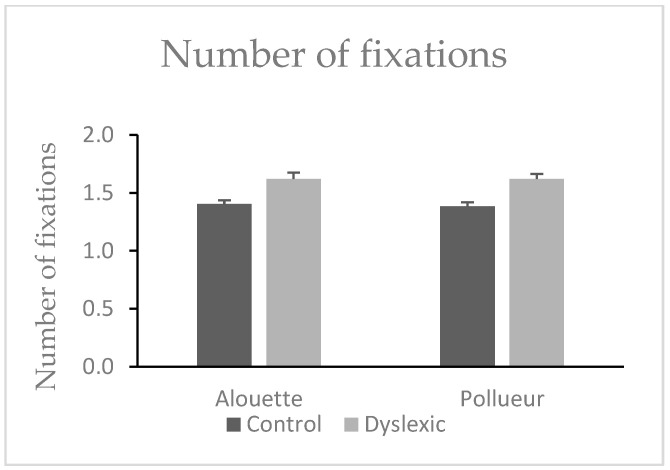
Mean number of fixations during reading the “Alouette” (meaningless) and the “Pollueur” (meaningful) text in the group of control and dyslexic participants. The values resent fixations following a pro-saccade. Error bars represent standard errors.

No significant difference was found between texts in the number of progressive fixations (Alouette: M = 1.51, SD = 0.23; Pollueur: M = 1.50, SD = 0.21), *W* = 472.00, *z* = 0.83, *p* = 0.413. However, a significant group effect was observed: dyslexic participants made more progressive fixations (M = 1.62, SD = 0.21) than control participants (M = 1.39, SD = 0.14), *U* = 77.00, *p* < 0.001, ΔM = 0.23 (see also Figure 2). The interaction was not significant, *U* = 212.00, *p* = 0.758.

#### 3.1.5. Fixation Duration (Fixations Associated with a Retro-Saccade)

There was no significant difference between texts (Alouette: M = 236.2 ms, SD = 40.2; Pollueur: M = 233.3 ms, SD = 41.2), *W* = 472.00, *z* = 0.83, *p* = 0.413. There was also no main effect of group, *U* = 180.00, *p* = 0.602. The interaction was not significant, *U* = 191.00, *p* = 0.820.

#### 3.1.6. Number of Fixations (Fixations Associated with a Retro-Saccade)

**Figure 3:**
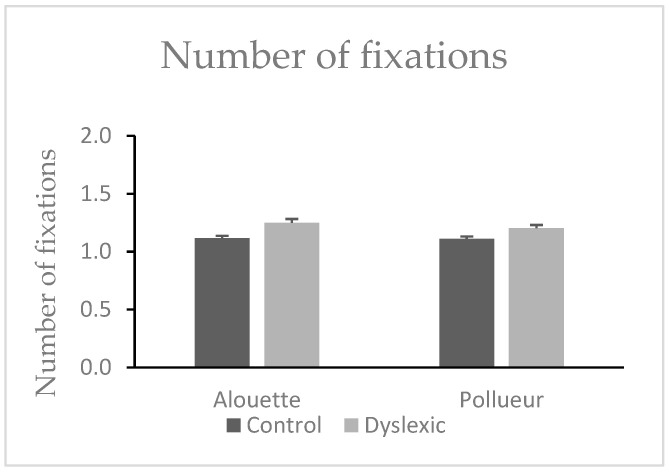
Mean number of fixations in the group of control and dyslexic participants during reading the “Alouette” (meaningless) and the “Pollueur” (meaningful) text. The values represent fixations following a retro-saccade. Error bars represent standard errors.

No significant text difference was found (Alouette: M = 1.18, SD = 0.14; Pollueur: M = 1.16, SD = 0.12), *W* = 512.00, *z* = 1.70, *p* = 0.090. Dyslexic readers made more regressive fixations (M = 1.23, SD = 0.13) than controls (M = 1.11, SD = 0.08), *U* = 81.00, *p* < 0.001, ΔM = 0.12 (see also Figure 3). The group × text interaction was not significant, *U* = 172.00, *p* = 0.461.

#### 3.1.7. Amplitude of Pro-Saccades

**Figure 4:**
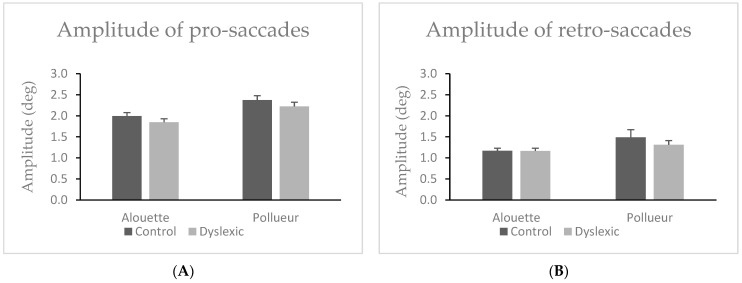
Amplitude of pro-saccades (**A**) retro-saccades (**B**) in the group of control and dyslexic participants during reading the “Alouette” (meaningless) and the “Pollueur” (meaningful) text. Error bars represent standard errors.

Participants made significantly shorter pro-saccades while reading the Alouette text (M = 1.92°, SD = 0.38) compared to the Pollueur text (M = 2.30°, SD = 0.46), *W* = 0.00, *z* = −5.51, *p* < 0.001, ΔM = –0.38° (see Figure 4A). There was no significant difference in pro-saccade amplitude between dyslexic readers (M = 2.04°, SD = 0.40) and controls (M = 2.18°, SD = 0.42), *U* = 241.00, *p* = 0.277. The group × text interaction was not significant, as both groups showed nearly identical amplitude differences between texts (Control: M = 0.38°, SD = 0.16; Dyslexic: M = 0.38°, SD = 0.18), *U* = 202.00, *p* = 0.968.

#### 3.1.8. Amplitude of Retro-Saccades

Participants made significantly shorter retro-saccades in the Alouette text (M = 1.17°, SD = 0.28) compared to the Pollueur text (M = 1.40°, SD = 0.65), *W* = 135.00, *z* = −3.70, *p* < 0.001, ΔM = −0.23° (see also Figure 4B). No significant difference in overall retro-saccade amplitude was observed between dyslexic (M = 1.24°, SD = 0.36) and control participants (M = 1.33°, SD = 0.48), *U* = 214.00, *p* = 0.718. The group × text interaction was not significant, *U* = 266.00, *p* = 0.076.

### 3.2. Analyses with Respect to Grammatical Category

#### 3.2.1. Fixation Duration (Associated with a Pro-Saccade)

No main effect (of Group [*F* < 1]; of Text [*F* < 1]; of Grammatical category [*F*(2,76) = 2.919, *MSE* = 2511.235, *p* = 0.071]) or interaction (Group by Text [*F*(1,38) = 1.591, *MSE* = 2285.748, *p* = 0.215]; Group by Grammatical category [*F* < 1]; Text by Grammatical category [*F* < 1]; Group by Text by Grammatical category [*F* < 1]) were found significant. This trend suggests a possible effect of grammatical category on fixation duration that did not reach significance, indicating that subtle differences may exist but require further investigation. Previous psycholinguistic studies often report differences in fixation duration across grammatical categories. The lack of significant effects in the present study may reflect specific characteristics of our adult dyslexic and control groups or task demands, suggesting that further research is needed to clarify these relationships.

#### 3.2.2. Number of Fixations (Associated with a Pro-Saccade)

**Figure 5:**
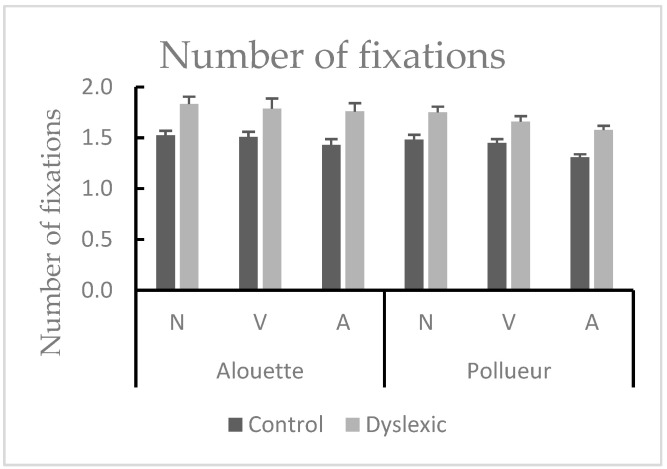
Mean number of fixations for nouns (N), verbs (V), and adjectives (A) during reading the “Alouette” (meaningless) and the “Pollueur” (meaningful) text in the group of control and dyslexic participants. The values represent fixations following a pro-saccade. Error bars represent standard errors. Results indicated more fixations for nouns than verbs and adjectives (N > V > A) across both groups and texts.

There was a main effect of Group [*F*(1,38) = 14.981, *MSE* = 0.307, *p* < 0.001, *η*^2^*p* = 0.283], since dyslexic participants made more fixations compared to the control group (mean difference = 0.3). Moreover, there was a main effect of Text [*F*(1,38) = 11.443, *MSE* = 0.047, *p* < 0.001, *η*^2^*p* = 0.261], with more fixations performed during reading the Alouette text compared to the Pollueur text. There was also a main effect of the Grammatical category [*F*(2,76) = 15.600, *MSE* = 0.407, *p* < 0.001, *η*^2^*p* = 0.291]. Post hoc tests revealed that there were more fixations on nouns compared to verbs (*p_holm_* = 0.047) and to adjectives (*p_holm_* < 0.001), and more fixations on verbs compared to adjectives (*p_holm_* = 0.002) (see Figure 5). However, interactions did not reach significance (Group by Text [*F* < 1]; Group by Grammatical category [*F* < 1]; Text by Grammatical category [*F*(2,76) = 2.058, *MSE* = 0.025, *p* = 0.145]; Group by Text by Grammatical category [*F* < 1]). Fixation patterns were stable across groups and texts, indicating no modulatory effect of grammatical category across conditions.

#### 3.2.3. Fixation Duration (Fixations Associated with a Retro-Saccade)

**Figure 6:**
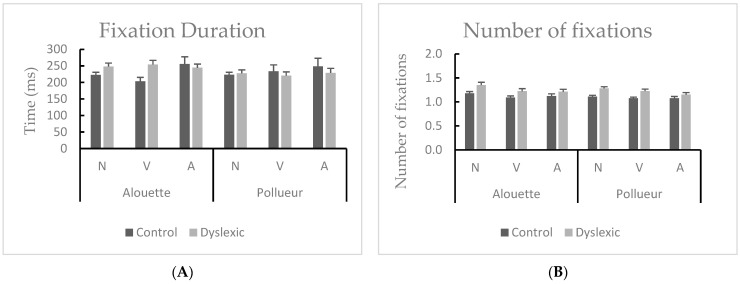
Mean of fixation duration (**A**) and of the number of fixations (**B**) for nouns (N), verbs (V), and adjectives (A) during reading the “Alouette” (meaningless) and the “Pollueur” (meaningful) text in the group of control and dyslexic participants. The values represent fixations following a retro-saccade. Error bars represent standard errors.

There was neither a main effect of Group [*F* < 1] nor a main effect of Text [*F*(2,76) = 6.779, *MSE* = 3373.983, *p* = 0.002]. There was only a significant main effect of Grammatical category [*F*(2,76) = 6.779, *MSE* = 2234.608, *p* = 0.004, *η*^2^*p* = 0.170]. Post hoc tests revealed that fixations associated with a retro-saccade were longer for nouns compared to adjectives (*p_holm_* = 0.017) and longer for verbs compared to adjectives (*p_holm_* = 0.002). With respect to interactions, there was a significant Group by Grammatical category interaction [*F*(2,76) = 7.505, *MSE* = 16771.789, *p* = 0.003, *η*^2^*p* = 0.185]. Post hoc tests revealed that fixations associated with a retro-saccade were longer for nouns compared to adjectives (*p_holm_* = 0.006) and longer for verbs compared to adjectives (*p_holm_* < 0.001) only in the control group (see Figure 6A). The other interactions did not reach significance (Group by Text [*F*(1,38) = 2.608, *MSE* = 3373.983, *p* = 0.116]; Text by Grammatical category [*F*(2,76) = 1.521, *MSE* = 2161.399, *p* = 0.435]; Group by Text by Grammatical category [*F* < 1]).

#### 3.2.4. Number of Fixations (Fixations Associated with a Retro-Saccade)

Repeated-measures ANOVA revealed a main effect of Group [*F*(1,33) = 12.545, *MSE* = 0.070, *p* = 0.001, *η*^2^*p* = 0.275]. Dyslexic participants made more fixations associated with a retro-saccade as compared to the control group (mean difference = 0.1). The main effect of Text did not reach significance [*F*(1,33) = 3.444, *MSE* = 0.021, *p* = 0.072], but the main effect of category was significant [*F*(2,66) = 5.234, *MSE* = 0.031, *p* = 0.009, *η*^2^*p* = 0.137]. Post hoc tests revealed that there were more fixations associated with a retro-saccade on nouns compared to verbs (*p_holm_* = 0.036) and to adjectives (*p_holm_* = 0.009) (see Figure 6B). However, interactions did not reach significance (Group by Text [*F* < 1]; Group by Grammatical category [*F*(2,66) = 1.248, *MSE* = 0.039, *p* = 0.292]; Text by Grammatical category [*F* <1]; Group by Text by Grammatical category [*F* < 1]).

### 3.3. Spearman’s Correlations

Lexical and oculomotor variables were computed at the word level. For each word, we calculated the mean number of fixations and mean fixation duration by averaging across all participants. These word-level measures were then correlated with lexical variables such as word frequency, length, and consistency.

#### 3.3.1. Pollueur Text (Meaningful Text)

There was a significant negative correlation between lexical frequency and the number of fixations associated with a progressive saccade in both the control (*r* = −0.572, *p* < 0.001) and dyslexic groups (*r* = −0.612, *p* < 0.001), indicating that higher frequency words elicited fewer fixations in both groups. Similarly, word length showed a significant positive correlation with the number of progressive fixations for both controls (*r* = 0.697, *p* < 0.001) and dyslexics (*r* = 0.752, *p* < 0.001), reflecting that longer words received more fixations. Additionally, in the dyslexic group only, word length was significantly positively correlated with the number of regressive fixations (*r* = 0.331, *p* < 0.001), suggesting that dyslexic readers tend to make more regressions on longer words, a pattern not observed in controls.

All other correlations, including those involving fixation durations and regressions in the control group, were not significant after applying the Bonferroni correction for multiple comparisons (adjusted significance threshold *p* < 0.00156). This indicates that word frequency and word length most robustly influence the number of progressive fixations, while word length uniquely influences regressions in dyslexic readers when reading the Pollueur text. The correlations related to the Pollueur text are shown in Table 3.

#### 3.3.2. Alouette Text (Meaningless Text)

Word frequency correlated negatively with the number of fixations (for fixations associated with a pro-saccade) in both the control (*r* = −0.319, *p* < 0.001) and the dyslexic group (*r* = −0.317, *p* < 0.001). The higher the frequency, the fewer fixations were needed to read the word.

Finally, the number of letters significantly correlated with the number of fixations in both the control (*r* = 0.753, *p* < 0.001) and the dyslexic group (*r* = 0.746, *p* < 0.001). The longer the word, the more fixations were needed in order to read it. Finally, no correlation was found to be significant between the consistency of graphemes to phonemes or of phonemes to graphemes and the other eye movement variables examined (duration and number of fixations associated with a pro- or a retro-saccade) (*p* > 0.05). Correlations for the Alouette text are presented in Table 4.

Overall, both meaningful (Pollueur) and meaningless (Alouette) texts showed that word-level features like frequency and length influence the number of progressive fixations—higher frequency words elicited fewer fixations, and longer words elicited more. However, only in the meaningful text, and only in the dyslexic group, was word length significantly linked to regressive fixations, which may reflect visual-attentional challenges and/or different language processing strategies.

### 3.4. Multiple Regressions

Multiple linear regressions were performed separately for control and dyslexic participants to test how word frequency, word length, grapheme-to-phoneme consistency (G→P), and phoneme-to-grapheme consistency (P→G) predicted four eye movement measures: fixation duration and number of fixations during forward saccades (pro-saccades) and regressions.

#### 3.4.1. Duration of Progressive Fixations

Significant models emerged only in controls for both texts (Alouette: R^2^ = 0.093, *p* = 0.009; Pollueur: R^2^ = 0.089, *p* = 0.002). In Alouette, both grapheme-to-phoneme (G→P) consistency (β = 0.220, *p* = 0.006) and phoneme-to-grapheme (P→G) consistency (β = 0.190, *p* = 0.016) predicted longer fixations. In Pollueur, higher word frequency (β = −0.283, *p* = 0.002) and greater G→P consistency (β = −0.265, *p* = 0.002) predicted shorter fixations. Dyslexic participants showed no significant predictors for either text (all *p* > 0.05). This contrast may reflect differences in task demands: Alouette requires exhaustive reading (encouraging detailed phonological processing), while Pollueur allows more lexical skipping, reducing fixation duration with easier or more consistent words.

#### 3.4.2. Number of Progressive Fixations

For the number of fixations in pro-saccades, strong and significant effects of word length were observed in both groups across both texts. In Alouette, the models were highly significant for controls (R^2^ = 0.570, *p* < 0.001) and dyslexics (R^2^ = 0.567, *p* < 0.001), with word length predicting more fixations in both groups (βs = 0.747 and 0.743, respectively, both *p* < 0.001). The same pattern held in Pollueur (controls: R^2^ = 0.507, *p* < 0.001, β = 0.660; dyslexics: R^2^ = 0.545, *p* < 0.001, β = 0.707), confirming that longer words consistently required more fixations, regardless of group or text.

#### 3.4.3. Duration of Regressive Fixations

For the duration of regressive fixations, no significant effects were found in either group for Alouette (all *p* > 0.05). In contrast, in Pollueur, the model reached significance only in dyslexics (R^2^ = 0.053, *p* = 0.049), with P→G consistency predicting longer regression fixations (β = 0.202, *p* = 0.007). While the model was not significant for controls (*p* = 0.137), G→P consistency still showed a notable effect (β = −0.190, *p* = 0.017), suggesting a divergence in how each group processes regressive movements based on phonological consistency.

#### 3.4.4. Number of Regressive Fixations

For the number of fixations associated with regressive saccades, significant models were found only in Pollueur, for both controls (R^2^ = 0.063, *p* = 0.027) and dyslexics (R^2^ = 0.092, *p* = 0.002). In controls, word frequency was the only significant predictor (β = 0.264, *p* = 0.009), with higher frequency words eliciting more regressive fixations. In dyslexics, word length predicted more regressions (β = 0.218, *p* = 0.020), suggesting that longer words triggered additional rereading. No significant effects were found in Alouette for either group (all *p* > 0.05). Table 5 summarizes regression results.

## 4. Discussion

To our knowledge, this is the first study to examine eye movements in dyslexic adults using an ecologically valid, text-reading paradigm while considering key linguistic parameters such as grammatical category, word length, lexical frequency, and orthographic consistency. This innovative approach allows us to investigate how dyslexia manifests during naturalistic reading, rather than isolated word or sentence tasks.

Our main findings indicate that, compared to control participants, adults with dyslexia exhibited longer reading times and a greater number of reading errors, especially in the Alouette (meaningless) text. They also produced more progressive and regressive fixations across both texts.

When examining grammatical categories, dyslexic participants made more fixations (both progressive and regressive), while in the control group, fixation durations associated with regressive saccades were longer for nouns compared to adjectives.

In the item-level analysis, distinct group effects emerged. More precisely, only in the dyslexic group did longer words trigger more regressive fixations. This finding was confirmed by multiple regression analyses, which showed that word length significantly predicted the number of regressive fixations only in the dyslexic group, whereas for progressive fixations, word length predicted behavior in both groups. Moreover, a group-specific consistency effect emerged: fixation durations were modulated by word consistency only in the control group. This suggests that controls may rely more effectively on consistent grapheme-to-phoneme mappings during reading, whereas dyslexic participants show reduced sensitivity to this phonological cue.

Additional findings, consistent across groups, revealed that the Alouette text prompted longer reading times, more errors, and more progressive fixations, whereas the Pollueur text elicited larger amplitude saccades. Across both groups, nouns elicited more fixations than verbs and adjectives (N > V > A) during both progressive and regressive saccades.

Finally, higher word frequency was associated with fewer and shorter progressive fixations, and longer words led to more progressive fixations. Notably, the frequency effect on fixation duration was observed only in the meaningful text.

### 4.1. Reading Time and Reading Errors

Dyslexic participants made more reading errors on the Alouette text, likely due to its lack of semantic coherence. Unlike meaningful texts, this type of material does not allow readers to use anticipation or inference strategies and requires strong decoding skills. As a result, individuals with dyslexia, who often have decoding difficulties, are more prone to errors when reading such text.

Importantly, our results show that dyslexic participants read more slowly than controls across both texts, regardless of whether the content was meaningful or meaningless. This indicates that semantic context, while helpful, does not eliminate the group difference in reading speed. The persistence of longer reading times highlights the ongoing difficulties dyslexic adults face, even after years of remediation and frequent exposure to reading in academic settings. These findings support previous research on the long-term nature of reading challenges in both children and adults with dyslexia.

### 4.2. Number of Fixations

One significant finding in our study was the increased number of progressive fixations. This result, typically observed in studies involving children [15,18,19,22,42,74], is now confirmed in French higher education students with dyslexia. Despite having undergone years of remedial education and being exposed to the demanding reading requirements of higher education, these individuals still tend to use a more serial decoding strategy during reading. This observation may be attributed to visual attentional deficits, supporting the hypothesis of a smaller visuo-attentional span in dyslexia, i.e., fewer letters processed simultaneously in a single fixation [10,14]. The significance of this effect across grammatical categories further supports this explanation. A similar result—an increased number of fixations in dyslexic adults— was observed in a study using a phonological lexical decision task with isolated stimuli [26]. Likewise, dyslexic German adults showed more frequent fixations during sentence reading [25]. The higher number of fixations observed in our study may suggest that, unlike control participants who make fewer fixations per word and may access the lexical route for whole word recognition through orthographic processing, dyslexic individuals may struggle to engage this route effectively. Instead, they might rely more on a serial sublexical decoding strategy based on grapheme-to-phoneme conversion rules [25].

### 4.3. Fixation Duration

An interesting result from our study concerns fixation duration. Fixation duration reflects the time spent processing visual information from a word during reading, including key processes such as grapheme-to-phoneme conversion. Our results indicated that, across both texts, fixation duration was similar between dyslexic and control participants, regardless of text difficulty. This contrasts with findings in dyslexic children, who typically show longer fixation durations than controls. In our adult sample, however, fixation durations were comparable, consistent with the results of Ward and Kapoula [75], who found similar fixation durations in 14-year-old dyslexic and non-dyslexic children when reading texts including Alouette.

Fixations are central to reading, as grapheme-to-phoneme conversion and lexical access occur during them. Therefore, similar fixation durations may reflect partial compensation in adult dyslexics, possibly due to improved decoding strategies or cumulative reading experience. Supporting this, previous studies have shown a relationship between reading proficiency and the efficiency of information gathering during fixation [76,77]. Seassau et al. [74] also observed that fixation duration shortens with age in dyslexic children, likely reflecting maturation of cortical systems involved in fixation control [78].

However, our results differ from those of a study on German adults, which reported longer fixation durations in dyslexics [25]. This discrepancy may be influenced by orthographic transparency, as German has more consistent grapheme-phoneme correspondences than French. It may also reflect sample differences—our participants were university students, likely exposed to more frequent and varied reading. It may also relate to methodological differences: we used longer texts and an ecologically valid task involving oral reading, whereas Hawelka et al. [25] sentence-level stimuli with silent reading. These factors could contribute to the improved fixation behavior observed in our sample. More research is needed to clarify these influences.

### 4.4. Consistency

In this study, consistency refers to the reliability of grapheme-to-phoneme correspondence, that is, how consistently a written letter or letter group maps onto a sound. This is important in dyslexia research because inconsistent mappings can increase decoding difficulty, especially for individuals with phonological processing deficits.

In control participants, grapheme-to-phoneme (G→P) and phoneme-to-grapheme (P→G) consistency significantly predicted fixation duration, but no such effects were observed in dyslexic participants. Specifically, in Alouette, more consistent G→P and P→G mappings led to longer fixations (possibly due to deeper phonological processing), whereas in Pollueur, more consistent G→P mappings and higher word frequency led to shorter fixations, suggesting easier decoding. On the contrary, dyslexic participants showed no significant effects of consistency on fixation duration in either text. This result may indicate that dyslexics appear less sensitive to phonological consistency, meaning they do not benefit from regular mappings between spelling and sound as controls do. This points to a core phonological processing deficit, consistent with established dyslexia theories. The absence of these effects could suggest impaired access to or use of grapheme-to-phoneme rules, even in adulthood.

A similar pattern was reported by Jones et al. [79], who found regularity effects in gaze durations during sentence reading only among non-dyslexic participants. Together, these findings suggest that individuals with dyslexia struggle to efficiently apply grapheme-to-phoneme rules, resulting in longer fixation durations regardless of consistency. This may reflect a floor effect, where phonological processing difficulties flatten the impact of consistency on reading behavior.

Phonological processing difficulties are also supported by results from phonological awareness tasks, in which dyslexic participants showed significantly lower performance, highlighting underlying phonological impairments.

### 4.5. Regressive Fixations

A key finding of our study is the significantly higher number of regressive fixations in dyslexic adults compared to controls, across both texts and grammatical categories.

This result, previously reported by several authors [24,75,79] supports the interpretation that dyslexic readers rely on serial decoding strategies, consistent with the dual-route model [80] and may also reflect a reduced visual attention span, as posited in the visual attention span theory [10].

The increased frequency of regressive fixations among dyslexic participants, observed across both texts, may reflect deficient visuo-attentional skills. This pattern was especially pronounced for longer words, as shown by a correlation between word length and the number of regressive fixations only in the dyslexic group. This suggests that as word length increases, so does the need to reread, highlighting a potential difficulty in processing complex orthographic input in a single pass.

Similar findings were reported by Ward and Kapoula [75] in French dyslexic children, though they found significant differences only with the meaningless Alouette text. This discrepancy may stem from differences in participant age and text difficulty, as their meaningful text may have been more accessible for their sample than in our study. These differences suggest that text complexity modulates the visibility of reading difficulties in dyslexia, linking them to higher linguistic deficits [15,81,82,83]. Nevertheless, the presence of increased regressions regardless of text type in our study also points to visual processing deficits, as indicated by a distinct oculomotor profile in dyslexic readers.

Multiple regression analyses further confirmed this pattern. Word length was a strong predictor of the number of progressive fixations in both groups, consistent with general reading effort. However, for regressive fixations, word length predicted behavior only in the dyslexic group. In the Pollueur text, longer words triggered significantly more regressions exclusively in dyslexic participants, reinforcing the idea that they require more rereading as word complexity increases.

In contrast to our findings, Hawelka et al. [25], reported no significant difference in the number of regressive saccades between dyslexic and control adults. While their dyslexic participants showed more progressive fixations of longer duration, our study found that dyslexic adults made more total fixations, similar in duration to controls, but also displayed more regressive fixations. A similar pattern—increased regressions without longer fixations—was also reported by Jones et al. [79] in English-speaking university students.

These differences may reflect the influence of orthographic transparency. In transparent languages like German, as in Hawelka et al. [25], dyslexic readers may rely on more efficient grapheme-to-phoneme mappings, leading to longer progressive fixations but fewer regressions. In less transparent languages like French or English, where such mappings are less consistent, dyslexic readers may adopt more regressive strategies to compensate, despite similar fixation durations.

These results may be interpreted through the lens of Grain Size Theory [84], which posits that readers of more transparent languages rely more heavily on serial grapheme-to-phoneme decoding, while readers of deeper orthographies tend to engage in more lexical, whole-word strategies. In line with this theory, our data suggest that French dyslexic readers, facing an inconsistent orthography, may attempt whole-word recognition but often fail, leading them to regress and refixate in order to decode the word more accurately [85].

Additionally, task differences may explain some variation. Hawelka et al. used discrete sentence reading, while our study presented participants with a continuous 16-line text, which may have increased the need for regressions. This highlights the importance of considering both language characteristics and task design when interpreting oculomotor patterns in dyslexia. Further cross-linguistic research is needed to disentangle these factors.

### 4.6. Secondary Results

Our secondary results, which do not focus on group differences, reveal patterns tied closely to text characteristics. The Alouette text, being meaningless and composed of infrequent words (particularly nouns), posed greater difficulty. This likely explains the increased reading time, more reading errors, and a higher number of progressive saccades, especially when examining grammatical categories. The need for a more serial, segmental reading strategy appears independent of the reader group. Similar findings were reported by Ward and Kapoula [75] using the same text with 14-year-old children, we replicated these results with adults. Conversely, the Pollueur text—being meaningful and more natural—elicited larger progressive and regressive saccades, consistent with previous research showing that easier texts result in longer saccade amplitudes [86].

Interestingly, we observed more progressive and regressive fixations on nouns across both texts, contradicting standard psycholinguistic findings that nouns are processed more easily [53,54,55,56,57,58]. This may be due to two factors: the Alouette text was deliberately constructed with infrequent words, particularly nouns, and the Pollueur text, drawn from a newspaper, reflected real-world linguistic variation without controlled grammatical distributions. As such, neither text was optimized to test word category effects, limiting generalization. Another explanation might be that the higher number of fixations on nouns could suggest greater lexical or syntactic processing demands compared to verbs or adjectives, consistent with prior psycholinguistic research [87,88].

We also found that, in both groups, more frequent and shorter words resulted in fewer fixations, in line with well-established effects in the eye movement literature [15,34,36,87,89]. Additionally, only in the Pollueur text did higher word frequency correspond to shorter fixation durations, likely because its natural content better captures real-life reading processes. These findings not only support established effects of word frequency and length but also underscore the ecological validity of studying eye movements in authentic texts.

## 5. Conclusions

This study investigated eye movement patterns in French university students with and without dyslexia during reading aloud two types of texts: one meaningful and one meaningless. We analyzed how specific linguistic parameters—grammatical category, word length, frequency, and consistency—influenced reading behavior.

Our results showed that dyslexic readers made more progressive and regressive fixations than controls, suggesting a reduced visuo-attentional span. The high number of progressive fixations in dyslexic participants may reflect reliance on sublexical processing through the activation of grapheme-to-phoneme conversion. Additionally, multiple regression analyses also revealed that word consistency significantly predicted fixation duration only in the control group. This suggests that non-dyslexic readers benefit from phonological regularities during reading, while dyslexic readers may be less sensitive to these cues, reflecting ongoing challenges in phonological processing.

Taken together, these findings support the coexistence of phonological and visuo-attentional deficits in dyslexia. Future cross-linguistic studies are essential to further understand how orthographic transparency influences reading strategies and eye-movement profiles in dyslexia. Such research could inform the development of targeted interventions that combine phonological training with strategies to enhance visuo-attentional span, ultimately supporting more effective remediation for individuals with dyslexia.

## Figures and Tables

**Table 1:** Assessment of reading and other cognitive functions of participants. Mean value (±standard deviation) for the different tests run for the two groups of participants (control readers, dyslexic readers). Significance levels are denoted as follows: ns = *p* > 0.10; *p* * = 0.01 < *p* < 0.05; p ** = 0.001 < *p* < 0.01; *p* *** = *p* < 0.001.

	Control Readers	Dyslexic Readers	Group Difference
Age (years)	21.9 ± 2.1	22.4 ± 3.1	ns
Similarities subtest WAIS IV (standard note)	10.8 ± 1.4	10.8 ± 2.3	ns
Matrices subtest WAIS IV (standard note)	10.8 ± 2.1	10.3 ± 1.9	ns
Regular word reading (score/20)	19.6 ± 0.6	18.6 ± 1.6	*p* *
Regular word reading (time in seconds)	11.2 ± 3.0	19.8 ± 7.4	*p* ***
Irregular word reading (score/20)	18.3 ± 1.9	17.5 ± 2.7	ns
Irregular word reading (time in seconds)	11.1 ± 3.1	18.7 ± 7.2	*p* ***
Pseudoword reading (score/20)	18.8 ± 1.7	16.1 ± 2.9	*p* ***
Pseudoword reading (time in seconds)	16.9 ± 5.3	32.9 ± 9.3	*p* ***
Initial phoneme deletion (score/10)	9.4 ± 0.7	6.9 ± 2.2	*p* ***
Initial phoneme deletion (time in seconds)	36.7 ± 10.8	55.9 ± 11.7	*p* ***
Spoonerisms (score/20)	18.7 ± 1.5	13.9 ± 5.0	*p* ***
Spoonerisms (time in seconds)	84.1 ± 25.5	232.3 ± 137.4	*p* ***
Non-word repetition (score/20)	19.4 ± 0.7	18.4 ± 1.3	*p* **
Non-word repetition (time in seconds)	71.1 ± 7.6	74.2 ± 14.3	*ns*
Rapid automatized naming (RAN) images (score/100) †	99.8 ± 0.5	98.4 ± 1.7	*p* ***
Rapid automatized naming (RAN) images (time in seconds)	67.8 ± 12.2	83.8 ± 11.8	*p* ***
Rapid automatized naming (RAN) numbers (score/100)	100.0 ± 0.2	99.6 ± 0.9	ns
Rapid automatized naming (RAN) numbers (time in seconds)	30.3 ± 9.5	52.6 ± 12.2	*p* ***
Rapid automatized naming (RAN) letter (score/50)	49.4 ± 1.1	49.6 ± 0.9	ns
Rapid automatized naming (RAN) letter (time in seconds)	15.1 ± 3.8	25.0 ± 6.8	*p* ***
Visuo-attentional span (score/100)	94.9 ± 4.3	79.4 ± 14.7	*p* ***

† The RAN scores represent the number of correct items named during the time taken to complete the task.

**Table 2:** Mean and standard deviation of reading time and errors, of duration and the number of fixations, after pro- and retro-saccades, and of the amplitude of pro- and retro-saccades for the two types of texts “Alouette” (meaningless) and the “Pollueur” (meaningful) in the group of control and dyslexic participants.

	Alouette		Pollueur	
	Control	Dyslexic	Control	Dyslexic
Reading time (s)	101.3 ± 15.2	141.4 ± 40.3	82.9 ± 11.7	108.1 ± 22.3
Reading errors	4.5 ± 3.2	12.8 ± 9.3	1.5 ± 1.1	3.8 ± 3.0
Fixation duration (ms) (fixations associated with a pro-saccade)	273.1 ± 44.9	273.2 ± 46.8	274.7 ± 34.7	269.0 ± 46.5
Number of fixations (fixations associated with a pro-saccade)	1.4 ± 0.2	1.6 ± 0.5	1.4 ± 0.2	1.6 ± 0.2
Fixation duration (ms) (fixations associated with a retro-saccade)	223.8 ± 34.0	248.6 ± 43.0	234.1 ± 40.6	232.5 ± 42.7
Number of fixations (fixations associated with a retro-saccade)	1.1 ± 0.1	1.3 ± 0.2	1.1 ± 0.1	1.2 ± 0.3
Amplitude of pro-saccades (deg)	2.0 ± 0.4	1.9 ± 0.4	2.4 ± 0.5	2.2 ± 0.5
Amplitude of retro-saccades deg)	1.2 ± 0.3	1.2 ± 0.3	1.5 ± 0.8	1.3 ± 0.5

**Table 3:** Correlations between the lexical properties of the words (frequency, number of letters, consistency of graphemes to phonemes and of phonemes to graphemes) and the different oculomotor parameters examined (number and duration of fixations associated with a pro- (PRO) and a retro-saccade (RETRO)) in the Pollueur text for the two groups of participants. Bold values indicate significant Spearman’s correlations.

POLLUEUER		Control		Dyslexics	
		*r*	*p*	*r*	*p*
Frequency	Fixation duration (PRO)	−0.181	0.013	−0.028	0.705
	Number of fixations (PRO)	**−0.572**	**<0.001**	**−0.612**	**<0.001**
	Fixation duration (RETRO)	0.132	0.085	0.151	0.043
	Number of fixations (RETRO)	−0.038	0.623	−0.191	0.010
Word length (Number of letters)	Fixation duration (PRO)	0.158	0.025	0.033	0.643
	Number of fixations (PRO)	**0.697**	**<0.001**	**0.752**	**<0.001**
	Fixation duration (RETRO)	−0.118	0.105	−0.089	0.221
	Number of fixations (RETRO)	0.146	0.048	**0.331**	**<0.001**
Consistency of graphemes to phonemes	Fixation duration (PRO)	−0.132	0.070	−0.165	0.023
	Number of fixations (PRO)	−0.118	0.105	−0.089	0.221
	Fixation duration (RETRO)	0.190	0.012	−0.070	0.346
	Number of fixations (RETRO)	0.014	0.860	−0.030	0.693
Consistency of phonemes to graphemes	Fixation duration (PRO)	−0.006	0.933	0.033	0.653
	Number of fixations (PRO)	−0.003	0.972	0.016	0.832
	Fixation duration (RETRO)	−0.053	0.487	0.093	0.212
	Number of fixations (RETRO)	0.009	0.904	−0.080	0.284

**Table 4:** Correlations between the lexical properties of the words (frequency, number of letters, consistency of graphemes to phonemes and of phonemes to graphemes) and the different oculomotor parameters examined (number and duration of fixations associated with a pro- (PRO) and a retro-saccade (RETRO)) in the Alouette text for the two groups of participants. Bold values indicate significant Spearman’s correlations.

ALOUETTE		Control		Dyslexics	
		*r*	*p*	*r*	*p*
Frequency	Fixation duration (PRO)	−0.141	0.092	−0.222	0.008
	Number of fixations (PRO)	**−0.672**	**<0.001**	**−0.764**	**<0.001**
	Fixation duration (RETRO)	−0.033	0.713	−0.217	0.011
	Number of fixations (RETRO)	−0.136	0.125	−0.208	0.309
Word length (Number of letters)	Fixation duration (PRO)	0.043	0.608	−0.020	0.813
	Number of fixations (PRO)	**0.753**	**<0.001**	**0.768**	**<0.001**
	Fixation duration (RETRO)	−0.083	0.350	0.063	0.464
	Number of fixations (RETRO)	0.139	0.117	0.284	0.160
Consistency of graphemes to phonemes	Fixation duration (PRO)	−0.185	0.027	0.041	0.628
	Number of fixations (PRO)	−0.030	0.724	−0.114	0.173
	Fixation duration (RETRO)	0.107	0.226	−0.067	0.434
	Number of fixations (RETRO)	0.180	0.042	0.037	0.856
Consistency of phonemes to graphemes	Fixation duration (PRO)	0.025	0.770	−0.054	0.518
	Number of fixations (PRO)	0.017	0.837	0.070	0.405
	Fixation duration (RETRO)	0.027	0.762	0.066	0.439
	Number of fixations (RETRO)	−0.063	0.475	−0.035	0.867

**Table 5:** Summary of multiple linear regression results for eye movement measures across texts and groups (C: Control; D: Dyslexic).

Measure	Group	Text	R^2^	Significant Predictors	β	*p*	Effect Direction
Duration of (pro)fixations	C	Alouette	0.093	G→P Consistency P→G Consistency	0.220 0.190	0.006 0.016	Longer fixations for high consistency
	C	Pollueur	0.089	Word Frequency G→P Consistency	−0.283 −0.265	0.002 0.002	Shorter fixations for high values
	D	Both	ns	None	—	>0.05	No effects
Number of (pro)fixations	C	Alouette	0.570	Word Length	0.747	<0.001	Longer words → more fixations
	C	Pollueur	0.507	Word Length	0.660	<0.001	Longer words → more fixations
	D	Alouette	0.567	Word Length	0.743	<0.001	Longer words → more fixations
	D	Pollueur	0.545	Word Length	0.707	<0.001	Longer words → more fixations
Duration of (retro)fixations	C	Alouette	ns	None	—	>0.05	No effects
	C	Pollueur	ns	G→P Consistency (marginal)	−0.190	0.017	Higher G→P consistency → shorter regressions
	D	Alouette	ns	None	—	>0.05	No effects
	D	Pollueur	0.053	P→G Consistency	0.202	0.007	Higher P→G consistency → longer regressions
Number of (retro)fixations	C	Alouette	ns	None	—	>0.05	No effects
	C	Pollueur	0.063	Word Frequency	0.264	0.009	More frequent → more regressions
	D	Alouette	ns	None	—	>0.05	No effects
	D	Pollueur	0.092	Word Length	0.218	0.020	Longer words → more regressions

## Data Availability

The datasets generated and/or analyzed during the current study are available from the corresponding author on reasonable request due to privacy reasons.
